# A new species of *Callispa* Baly (Coleoptera, Chrysomelidae, Cassidinae, Callispini) infesting coconut palm ( *Cocos nucifera* L.) in India

**DOI:** 10.3897/zookeys.269.4240

**Published:** 2013-02-14

**Authors:** K. M. Shameem, K. D. Prathapan

**Affiliations:** 1 Department of Entomology, Kerala Agricultural University, Vellayani P.O., Trivandrum - 695 522, Kerala, India

**Keywords:** Chrysomelidae, coconut, *Callispa*, new species, host plants, insect pest, India

## Abstract

*Callispa keram* **sp. n.** infesting coconut palm (*Cocos nucifera* L.) in Kerala, India is described and illustrated. *Livistona chinensis* R.Br. and *Syagrus* *romanzoffiana* (Cham.) Glassman are reported as additional host plants.

## Introduction

The coconut palm, *Cocos nucifera* L. is an important source of food and vegetable oil and is intimately associated with the social and cultural heritage of the people in Asia and Oceania (Menon and Pandalai 1958, Thampan 1993). India is one of the largest producers of coconut in the world. Mariau (2004) reviewed the biology of leaf beetles infesting the oil palm, *Elaeis guineensis* Jacq. and the coconut palm, documenting 79 species of Chrysomelidae on these two palms. The hispine cassidinae with 59 species in 37 genera are the most numerous leaf beetles on oil and coconut palms worldwide and include such serious pests as *Brontispa longissima* Gestro and *Promecotheca cumingii* Baly. Nair and Oommen (1965) reported the occurrence of an unspecified species of *Callispa* Baly 1858, on coconut palm in Kerala, India.

The genus *Callispa* comprises about 165 species distributed in the Oriental and Afrotropical Regions (Staines 2011). Trophic selections of *Callispa* are confined to the monocot families Araceae, Arecaceae, Cyperaceae, Musaceae, Poaceae, Orchidaceae and Zingiberaceae (Uhmann 1969; Jolivet and Hawkeswood 1995; Reid 1998; Schöller 2007, 2008; Staines 2011; Lee et al. 2012). Information on the immature stages of the genus is fragmentary and is limited to nine species (Lee et al. 2012). Studies on life history include Chen (1929), Nair and Oommen (1965), Zaitsev (2001) and Lee et al. (2012). Eggs, deposited singly (Nair and Oommen 1965, Lee et al. 2012) or in small groups (Chen 1929, Lee et al. 2012), are enclosed in a membranous ootheca. Both larvae and adults are open feeders on leaves and pupation occurs on the leaf itself. In India, the genus *Callispa* is represented by 32 species (Weise 1913;Maulik 1919, 1923; Uhmann 1927; Pic 1937, 1943; Medvedev 1993; Basu 1999; Staines 2011) while several others await naming and description.

Nair and Oommen (1965) is the only report of the genus *Callispa* on coconut palm. In this paper, we report our investigations on this coconut *Callispa* and describe it as a new species.

## Material and methods

Coconut is raised on a large scale both in homesteads as well as plantations in coastal and midland regions of Kerala. The presence of adults and larvae of the coconut *Callispa* is indicated by the characteristic feeding damage and could be easily collected from the abaxial side of leaflets. Specimens were collected from the plains of southern, central and northern Kerala. Attempts were also made to check its presence in the high range regions of Kerala as well as the dry coconut growing tracts of Tamil Nadu, adjoining Kerala. Besides coconut, other palms were also searched for the feeding damage and life stages of the species. Observations on the biology were carried out in the field as well as in the laboratory at the Vellayani campus of the Kerala Agricultural University.

Descriptive terminology follows Chaboo (2007). The holotype of the new species is deposited in the Natural History Museum, London (BMNH). Paratypes will be deposited in the Natural History Museum, London, Museo Civico di Storia Naturale, Genova, Italy (MCSN), National Bureau of Agriculturally Important Insects, Bengaluru, India (NBAII), National Pusa Collection, Indian Agricultural Research Institute, New Delhi, India (NPC), University of Agricultural Sciences, Bengaluru, India (UASB), National Museum of Natural History, Smithsonian Institution, Washington, DC, USA (USNM), and in the personal collection of the authors (PKDC). Non-type material are also deposited in the University of Kansas Insect Collection (SEMC). Plant vouchers of *Livistona chinensis* R.Br. (Accession no. 6697) and *Syagrus* *romanzoffiana* (Cham.) Glassman (Accession no. 6698) are deposited in the Calicut University Herbarium, Calicut, India. In the descriptions below, a forward slash (/) separates different lines on data labels.

## Results

### Generic diagnosis of *Callispa* Baly

Adults are oblong ovate, neither spiny nor tuberculate, 3–10 mm long, flat to moderately convex beetles. Other salient features of the genus are head narrowly produced between antennae; pronotum quadrate, broader than long, anterolateral angles rounded, anterior trichobothrium absent, disc shallowly depressed on either side; elytron with ten rows of punctures and a short scutellar row; claw tarsomere small, hardly extending beyond setae on ventral side of bilobed third tarsomere; upper border of mouth cavity in close proximity to antennal sockets; and scutellum quadrate with rounded posterior margin.

#### Callispa keram

Shameem & Prathapan sp. n.

urn:lsid:zoobank.org:act:2AD413D6-160B-4911-81CE-8F9A8B6FB863

http://species-id.net/wiki/Callispa_keram

Figs 1 [Fig F2] –14

##### Description of adult.

**Figures 1–3.**
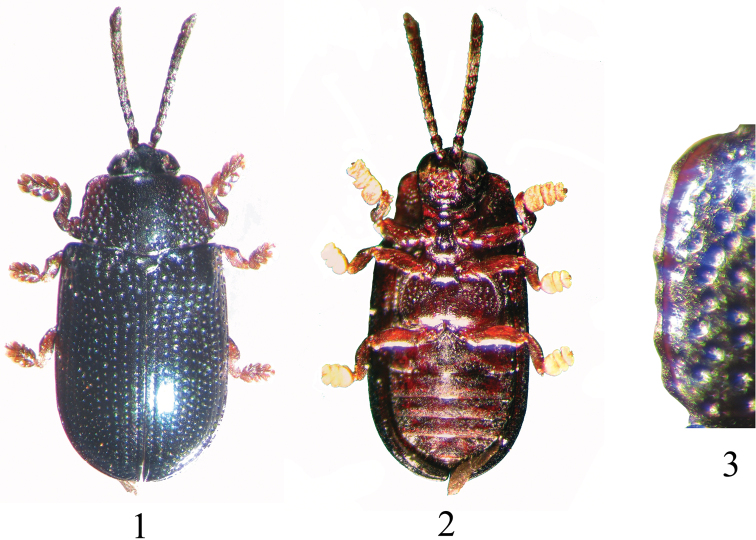
*Callispa keram* sp. n. **1** dorsal habitus **2** ventral habitus **3** lateral margin of pronotum.

Length 3.36–4.32 mm, width 1.73–2.35 mm. Vertex metallic black with blue tint; frontoclypeus black; gena, gula piceous to dark rufous brown; mouth parts dark rufous brown with labrum distinctly darker. Antenna piceous to dark rufous brown, often with proximal antennomeres darker than distal. Pronotal disc metallic black with blue tint, and turning rufous brown towards lateral margin in many specimens. Scutellum metallic black with blue tint. Elytra entirely dark metallic blue (Fig. 1). Venter and legs entirely dark rufous brown (Fig. 2).

Vertex minutely punctate, surface finely reticulate; midfrontal sulcus absent. Midcranial sulcus present as shallow indistinct groove evident anteriorly and posteriorly. Post-callinal transverse depression deeply impressed. Last maxillary palpomere as long as or longer than preceding two combined. Scape a little longer than half of pedicel; length ratio of antennomeres 2–11 equals 1.00 : 0.93-1.07 : 0.73-0.78 : 0.73-0.78 : 0.60-0.71 : 0.67-0.71 : 0.64-0.67 : 0.67-0.78 : 0.71-0.73 : 1.35-1.50.

Pronotum 1.53–1.67 times wider than long; posteriorly 1.14–1.20 times wider than anteriorly. Disc distinctly raised along middle 1/3, with transverse depression near posterior margin in front of scutellum. Disc impunctate anteriorly in middle as well as along a narrow mid-line; rest of raised middle portion with scattered small and minute punctures. Disc on either side of raised middle area moderately depressed with deep, large, circular punctures; distance between punctures less than diameter of individual puncture. Lateral margin anteriorly as broad as posteriorly, prominently scalloped with four to six emarginations (Fig. 3). Scutellum broader than long, convex on top, very minutely punctate and reticulate.

Elytron with shallow post-basal transverse depression deeper laterally; elytral apex convex. Scutellar row short, with three to five punctures; additional sixth row of punctures arises from middle of elytron, forming eleven rows of punctures, excluding scutellar row, present just behind middle. Distance between adjacent rows more than diameter of one puncture in middle of disc; distance between adjacent punctures in one row variable. Interstices flat, extremely minutely punctate. Punctures large, deep, round to oval. Hypomeron of pronotum with large, shallow punctures, punctures absent towards lateral margin, denser towards tergosternal suture. Prosternal intercoxal process channelled near margins on four sides, convexly raised along middle on top. Metasternum with granulate area bearing number of large, round, deep punctures anteriorly on either side, posterior to mesocoxal cavity.

**Figures 4–10. F2:**
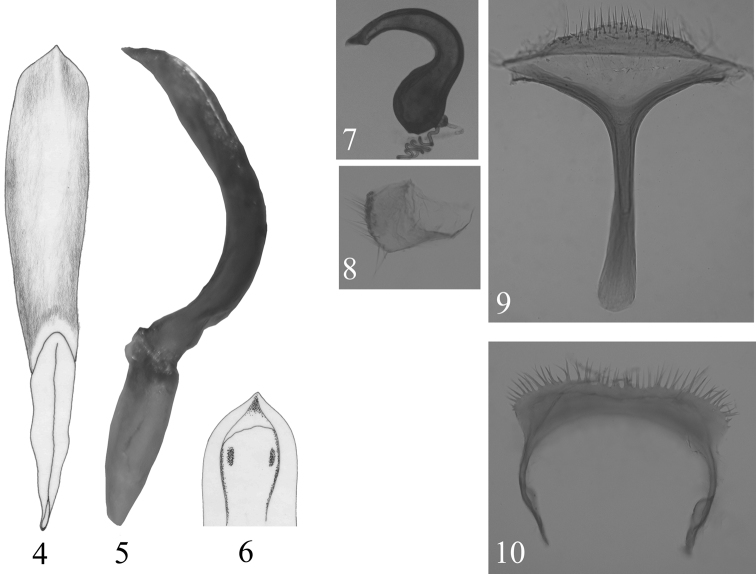
*Callispa keram* sp. n. **4** median lobe of aedeagus, ventral view **5** median lobe of aedeagus, lateral view **6** median lobe of aedeagus, distal opening **7** spermatheca **8** coxite **9** sternite VIII **10** tergum IX.

Aedeagus with basal piece poorly sclerotized. In lateral view, strongly curved near middle, apical 1/3 almost straight, apex acutely pointed (Fig. 5). Ventral side convex, with a sharply raised ridge along middle of distal region, with shallow depression on either side of ridge (Fig. 4). Apical foramen partially covered with a lamina bearing sclerotized plate on either side (Fig. 6). Arms of tegmen subequal to stem.

Receptacle of spermatheca longer than wide with inner side strongly convex, outer side gently concave; pump strongly curved, not differentiated from receptacle, about twice as long as receptacle, apical appendix acute, well developed; duct and gland inserted separately (Fig. 7). Sternite VIII with convex distal margin bearing numerous setae (Fig. 9). Spiculum gastrale anteriorly as wide as posteriorly. Coxite broader than long with setae along posterior margin (Fig. 8). Tergum IX represented by horseshoe shaped single sclerite with bristles along posterior margin (Fig. 10).

No apparent sexual dimorphism, except for slightly larger females (3.69–4.32 mm) compared to males (3.36–3.79 mm).

##### Etymology.

The specific epithet *keram*, literally means coconut in Malayalam, the language of Kerala, the southern Indian state where the insect occurs. It refers to the host plant as well as the type locality, *Keralam*, the land of coconut.

##### Types.

Holotype ♂, with labels as follows: 1) India: Kerala / Vellayani / 08°25'47.5"N, 76°59'8.3"E/ 9.ii.2012 18 m / Shameem K. Coll. / *Ex* Coconut (white label). 2) HOLOTYPE / *Callispa keram* sp. n. / des. Shameem & Prathapan, 2012 (red label) (BMNH).

Paratypes (99 specimens, all specimens with a white locality label as given below, besides a second pink label: PARATYPE / *Callispa keram* sp. n. / des. Shameem & Prathapan, 2012): 5 unsexed. the same labels as for holotype; 1 ♀, 1 unsexed. same data as for holotype except date 8.i.2012; 1 unsexed. same data except date 19.i.2012; 6 unsexed. same data except date 24.i.2012; 1 ♀. same data except date 27.i.2012; 8 unsexed. same data except date 2.ii.2012; 2 unsexed. same data except date 9.ii.2012; 4 unsexed. same data except date 16.ii.2012; 3 unsexed. same data except date 21.ii.2012; 1 ♀, 1 unsexed. same data except date 22.ii.2012; 8 unsexed. India: Kerala / Vellayani / 08°25'47.5"N, 76°59'8.3"E/ 12.xii.2011 18 m / Shameem K. Coll. / *Ex Livistona*; 1 ♀, 1 unsexed. same data except date 22.xii.2011; 2 ♂, 1 ♀, 1 unsexed. same data except date 2.ii.2012; 1 unsexed. same data except date 9.ii.2012; 1 unsexed. India: Kerala / Vellayani / 08°25'47.5"N, 76°59'8.3"E/ 9.i.2012 18 m / Shameem K. Coll. / *Ex Syagrus*; 1 ♀. same data except date 21.i.2012; 2 ♂, 1 ♀, 9 unsexed. India: Kerala / Vellayani / 08°25'47.5"N, 76°59'8.3"E/ 6.i.2012 18 m / Shameem K. Coll.; 3 ♂, 6 unsexed. same data except date 7.i.2012; 1 ♂, 1 ♀. same data except date 8.i.2012; 2 unsexed. same data except date 9.i.2012; 1 ♀, 1 unsexed. same data except date 11.i.2012; 2 ♂, 3 unsexed. same data except date 12.i.2012; 2 unsexed. same data except date 17.i.2012; 1 ♂. same data except date 19.i.2012; 1 unsexed. same data except date 24.i.2012; 1 unsexed . India: Kerala / Vallamkulam / 25.xii.2011 / Prathapan KD Coll. / *Ex* Coconut; 1 ♀. same data except locality Pandanad; 1 ♀, 1 unsexed. India: Kerala / Tirurangadi / 25.xii.2011 / Shameem K. Coll. / *Ex* Coconut; 2 unsexed. India: Kerala / Calicut University / 26.xii.2011 / Shameem K. Coll. / *Ex* Coconut; 1 unsexed. India: Kerala / Tirurangadi / 25.xii.2011 / Shameem K. Coll. / *Ex Livistona*; 1 unsexed. same data except date 6.ii.2012.

(4 specimens with the following labels, besides a pink label: PARATYPE / *Callispa keram* sp. n. / des. Shameem & Prathapan, 2012) 1 unsexed. 1) On Coconut / Vellayani / 26-8-56 / M.R.G.K.N. 2) 2. 3) Z.S.I. / Lot No. 47 / 1956; 1 unsexed. 1) 2. 2) Z.S.I. / Lot No. 47 / 1956; 1 unsexed. 1) 2. 2) Z.S.I. / Lot No. 47 / 1956. 3) *Callispa* sp / nr. *minima /* gestro / S.P. Shukla det ’57; 1 unsexed. 1) 5. 2) ? Callispa sp. / R. N. Mathur det. (10 BMNH, 5 MCSN, 10 NBAII, 48 NPC, 3 PKDC, 13 UASB, 10 USNM).

##### Distribution.

India (Kerala).

##### Remarks.

*Callispa keram* sp. n. can be differentiated from the other metallic black or blue species of *Callispa* in southern India, by the shape of the distinctly scalloped lateral margin of the pronotum (Fig. 3). Other southern Indian species with metallic black or blue dorsum, namely, *Callispa coerulodorsata* Maulik, *Callispa minima* Gestro and *Callispa violaceicornis* Pic have straight or evenly curved lateral pronotal margin. *Callispa keram* closely resembles *Callispa minima* in having shiny blue black dorsum and brown venter, besides being more or less similar in size. However, they can be separated based on the shape of the lateral margin of the pronotum as well as the finely rugose interstices on the basal portion of the elytron (elytral interstices are smooth in *Callispa keram*). In *Callispa coerulodorsata*, the ventral side is black and the scutellum bears three characteristic deep notches radiating from the centre, however, the ventral side in *Callispa keram* is rufous brown and the radiating notches on the scutellum are absent. The pronotum is strongly narrowed anteriorly in *Callispa violaceicornis*, while it is weakly narrowed towards front in the new species. Metallic blue-black species of southern Indian *Callispa* can be separated using the key given below.

##### Host plants.

Arecaceae: *Cocos nucifera* L., *Livistona chinensis* R.Br. (Chinese fan palm or fountain palm) and  *Syagrus* *romanzoffiana* (Cham.) Glassman (Queen palm).

##### Biology.

**Figures 11–16.**
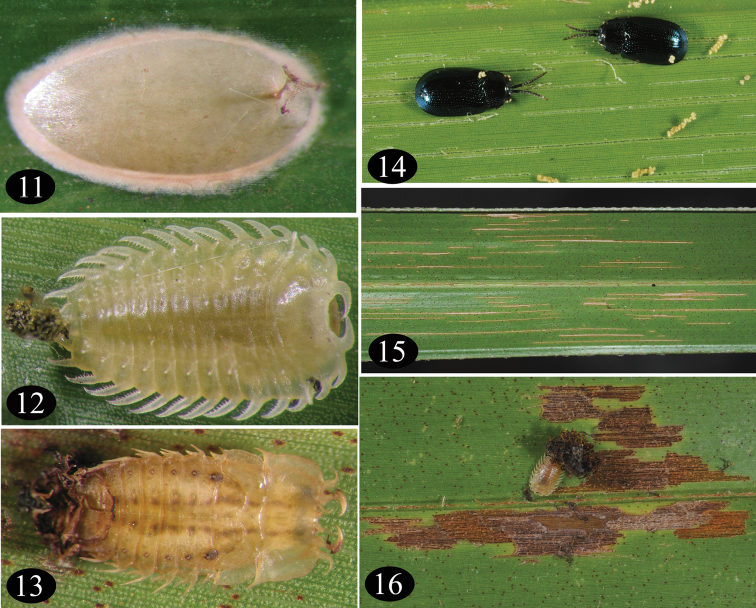
*Callispa keram* sp. n. **11** ootheca **12** larva **13** pupa **14** adult **15** adult feeding trough **16** larval feeding trough.

Nair and Oommen (1965) studied the biology and figured the life stages of *Callispa keram*. All the life stages (Figs 11–14) are confined to the abaxial side of the leaflets. According to Nair and Oommen, the beetles mate 5–7 days after emergence and commence oviposition thereafter. A female lays 28–54 eggs during an oviposition period of 46–113 days and the life cycle is completed in 34–43 days. Eggs are deposited singly in simple ootheca (Fig. 11). Attempts to separate the eggs from the ootheca resulted in rupture of the eggs. Feeding troughs of adults appear as characteristic narrow lines (Fig. 15) on the leaves while that of the larvae appear as brownish irregular patches (Fig. 16). All life stages of *Callispa keram* were observed on all three known host plants. *Callispa keram* was not observed in the high altitude regions of Kerala as well as in the dry coconut growing tracts of Tamil Nadu, adjoining Kerala.

### Key to metallic blue-black species of *Callispa* in southern India

**Table:** 

1	Scutellum with three deep radiating notches from the centre: one to the apex and others to the basal angles; abdominal ventrites black	*Callispa coerulodorsata* Maulik
–	Scutellum without deep radiating notches; abdominal ventrites rufous brown to dark brown	2
2(1)	Pronotum strongly narrowed anteriorly, posteriorly 1.5 times wider than anteriorly; length 5.0 mm	*Callispa violaceicornis* Pic
–	Pronotum weakly narrowed anteriorly, posteriorly 1.1–1.2 times wider than anteriorly; length 3.3–4.4 mm	3
3(2)	Lateral pronotal margin prominently scalloped with four to six emarginations; elytral interstices smooth throughout	*Callispa keram* sp. n.
–	Lateral pronotal margin straight, not scalloped, without emarginations; elytral interstices rugose basally	*Callispa minima* Gestro

## Discussion

Mariau (2004) divided the hispines associated with coconut and oil palms into two ecological groups: leaf-browsing and leaf-mining. *Callispa keram* belongs to the former group. While *Callispa keram* is a minor pest of little economic significance, the possibility of it becoming a significant pest, with change in biotic and abiotic factors, cannot be ruled out. Given the economic implications of the hispine pests of coconut palm such as *Brontispa longissima* and *Promecotheca cumingii*, *Callispa keram* is important as a potential invasive pest in other coconut growing countries far away from its native habitat. This is the first report of *Callispa keram* on *Livistona chinensis* and *Syagrus* *romanzoffiana*, two exotic palms introduced into India as ornamental plants.

## Supplementary Material

XML Treatment for Callispa keram
